# Efficacy and Safety of 0.03% Atropine Eye Drops in Controlling Myopia Progression: A One-Year Prospective Clinical Study

**DOI:** 10.3390/jcm13113218

**Published:** 2024-05-30

**Authors:** Dovile Simonaviciute, Andrzej Grzybowski, Arvydas Gelzinis, Reda Zemaitiene

**Affiliations:** 1 Department of Ophthalmology, Medical Academy, Lithuanian University of Health Sciences, 44037 Kaunas, Lithuania; arvydas.gelzinis@lsmu.lt (A.G.); reda.zemaitiene@lsmu.lt (R.Z.); 2 Department of Ophthalmology, University of Warmia and Mazury, 10-724 Olsztyn, Poland; ae.grzybowski@gmail.com; 3 Institute for Research in Ophthalmology, 60-554 Poznan, Poland

**Keywords:** low-concentration atropine, myopia management, myopia progression, low-dose atropine, myopia control

## Abstract

**Objective:** To investigate the efficacy and safety of one-year treatment with 0.03% atropine eye drops for slowing myopia progression among children aged 6–12 years. **Methods:** Healthy Caucasian children aged 6–12 years with cycloplegic spherical equivalent (SE) from −1.0 D to −5.0 D and astigmatism and anisometropia ≤1.5 D were included. Changes in mean axial length (AL) and objective SE as well as changes in intraocular pressure (IOP), central corneal thickness (CCT), anterior chamber depth (ACD) and lens thickness (LT) were assessed in the 0.03% atropine eye drops group and the control group from baseline through the 1-year follow-up. The proportion of participants showing myopia progression of <0.5 D from baseline in each group and any potential side effects in 0.03% atropine group were evaluated. **Results:** The study involved 31 patients in the 0.03% atropine eye drops group and 41 in the control group. Administration of 0.03% atropine for 1 year resulted in a mean change in SE of −0.34 (0.44) D/year, significantly lower than the −0.60 (0.50) D/year observed in the control group (*p* = 0.024). The change in AL was 0.19 (0.17) mm in the 0.03% atropine group, compared to 0.31 (0.20) mm in the control group (*p* = 0.015). There were no significant differences in changes of IOP, CCT and LT between the groups (all *p* ≥ 0.05). The 0.03% atropine group had a significantly greater increase in ACD compared to the control group (*p* = 0.015). In total, 64.5% of patients in the 0.03% atropine group showed progression <0.5 D/year, in contrast to 39.0% in the control group (*p* = 0.032). Adverse events were reported in 13 (35.0%) out of 37 patients in the treatment group, leading to discontinuation of the eye drops in six (16.0%) cases. None of the adverse events were severe. **Conclusions:** Despite a higher incidence of adverse events, 0.03% atropine eye drops effectively slowed the progression of myopia over 1-year.

## 1. Introduction

In recent decades, myopia has become a prominent cause of visual impairment worldwide, experiencing a rapid increase in prevalence [1]. It is predicted that approximately half of the world’s population will be myopic by 2050, of which 10% will have a high degree of myopia [1]. High myopia presents a significant risk factor for severe complications, including retinal detachment, myopic choroidal neovascularization, myopic macular degeneration, foveoschisis, glaucoma and cataract [2]. The likelihood of these complications increases with each additional diopter of myopia [3]. As a result, there has been a growing emphasis on controlling myopia progression in recent ophthalmic research.

Various approaches have been explored to slow down myopia progression, including spectacle lenses, dual-focus lenses, orthokeratology contact lenses, repeated low-level red-light therapy and atropine eye drops [4,5,6]. Among the strategies available for managing myopia progression, atropine eye drops are widely regarded as one of the most effective interventions, with numerous studies demonstrating their ability to effectively and safely manage myopia progression in children, both those with premyopia and those already myopic [7,8,9,10,11]. However, it is important to note that the efficacy of atropine is concentration-dependent, with higher concentrations associated with increased risk of adverse events and rebound effects [11,12,13]. Post cessation of atropine eye drops, myopia tends to progress at a faster rate compared to its progression during treatment with atropine eye drops [14,15]. Nevertheless, the optimal treatment regimen for atropine, particularly concerning dosage and duration has yet to be definitively established. Despite the widespread use of 0.01% atropine as the safest concentration for controlling myopia progression, conflicting evidence in the literature has emerged due to variations in treatment response [11,16,17,18,19]. For example, the results of a recent clinical trial showed that 0.01% atropine eye drops did not reduce changes in the spherical equivalent (SE) or axial length (AL) among children in the United States [16]. The Low-Concentration Atropine for Myopia Progression (LAMP) phase 2 study suggested that 0.05% atropine eye drops may be the optimal concentration for slowing myopia progression, although it has been noted to cause more pronounced side effects in Caucasian children compared to Asian children [10,20]. While most studies investigating the effects of atropine eye drops on myopia progression have been conducted in Asian populations, studies in non-Asian countries often include participants from diverse ethnic backgrounds [21,22,23]. Consequently, while the efficacy and safety profile of low-dose atropine has been well-documented in Asian children, its effects in Caucasian populations remain less explored. A recent meta-analysis exploring the efficacy and safety of atropine in myopic children suggested that atropine eye drops may be more effective in Chinese children compared to those from other regions [11]. On the contrary, a study conducted in the Netherlands by Polling et al. [21] demonstrated the effectiveness of 0.5% atropine eye drops in controlling myopia progression, irrespective of the ethnicity. While the study primarily involved European children (68.8%), it also included 23.4% Asian subjects. However, nearly 83% of the treated children reported experiencing side effects from the 0.5% atropine eye drops. Therefore, the identification of an optimal concentration, considering the risk-benefit ratio, for Caucasian children still remains imperative.

The present study aimed to investigate the efficacy and safety of 1-year treatment with 0.03% atropine eye drops for reducing myopia progression in Caucasian children aged 6–12 years.

## 2. Materials and Methods

A prospective study was conducted to assess the efficacy and safety of 0.03% atropine eye drops at the Department of Ophthalmology, Hospital of Lithuanian University of Health Sciences, Kaunas clinics, from March 2021 to January 2024. Informed consent forms were signed by at least one parent or legal guardian of all participants and either written or verbal assent was obtained from the children involved. The study complied with Good Clinical Practice standards. The study procedures were conducted in accordance with the principles outlined in the Declaration of Helsinki and the approval to conduct the study was granted by the Lithuanian Bioethics Committee (Approval No. BE-2–18).

### 2.1. Study Participants

The study enrolled Caucasian children aged 6 to 12 years with a cycloplegic SE (sphere plus half of the cylinder power) refraction ranging from −1.0 D to −5.0 D as well as astigmatism and anisometropia of 1.5 D or less. Exclusion criteria included prior use of atropine eye drops or other methods for myopia progression control, congenital or chronic ocular diseases (e.g., cataracts, congenital retinal diseases, amblyopia, strabismus), anisometropia or astigmatism exceeding 1.5 D, a history of ocular surgeries, and systemic conditions such as cardiac, endocrinological and respiratory diseases or chromosomal abnormalities.

### 2.2. Study Interventions

All children diagnosed with myopia were advised on treatment options for myopia progression control. Patients and their parents or legal guardians received information about the available methods for managing myopia progression and those who decided to begin treatment with atropine eye drops were invited to participate in the study. Participants in the atropine group consented to self-administer 0.03% atropine eye drops once nightly in both eyes for a duration of 1 year. The corresponding control group was formed from the patients who agreed to participate in the study but declined treatment for myopia progression. Notably, patients were responsible for purchasing the atropine eye drops at their own expense from a pharmacy.

### 2.3. Study Procedures

All myopic children had a comprehensive ophthalmological examination at both the baseline and the 12-month follow-up visits. Following the initial visit, patients were actively encouraged to attend follow-up appointments through the phone calls and re-examination invitations to ensure consistent monitoring of their ocular health. Only measurements from the right eye were included in the analysis [24]. Cycloplegic refraction and intraocular pressure (IOP) were evaluated in each eye using the autorefractometer (Auto Ref/Kerato/Tono/Pachymeter TonoRef, III Nidek, Gamagori, Japan) [25,26]. Cycloplegia was induced by administering 1% cyclopentolate twice, with a 5 min interval between doses, followed by refraction assessment 20 min after the second drop [27]. A detailed examination of the anterior eye segment and the fundus was conducted using a slit lamp microscope. Ocular AL, anterior chamber depth (ACD), lens thickness (LT) and central corneal thickness (CCT) were measured using the partial coherence interferometer IOLMaster 700 (Carl Zeiss Meditec AG, Jena, Germany) before cycloplegia [28]. At visits, participants and their parents were encouraged to disclose any side effects and medical conditions experienced since the last visit. All adverse events were recorded, including symptoms such as allergy-related reactions, photophobia, blurred near vision or visual impairment. Additionally, single vision spectacle lenses were prescribed to all the patients and new prescriptions were provided as needed to ensure appropriate best corrected visual acuity.

### 2.4. Outcomes

The primary outcome of the study was to analyze the changes in mean AL and cycloplegic SE from the baseline visit to the 1-year follow-up period in both the 0.03% atropine and control groups. These parameters served as key indicators to evaluate the rate of myopia progression during the study duration. Secondary outcomes included assessing changes in IOP, CCT, ACD and LT in both the control and 0.03% atropine groups. Additionally, the study aimed to analyze the proportion of participants showing myopia progression of less than 0.5 D from baseline within each group. Adverse events were assessed based on reported complaints by either the parents or legal guardians or the children participating in the study. During visits, children and their parents or legal guardians were encouraged to openly discuss any side effects or medical conditions experienced while using 0.03% atropine eye drops. Specific inquiries were made regarding symptoms such as dilated pupils, heightened sensitivity to light or blurred near vision.

### 2.5. Statistical Analysis

Statistical analyses were conducted using SPSS version 29.0.1.0 (IBM Corp. Released 2023. IBM SPSS Statistics for Windows, Version 29.0.2.0 Armonk, NY, USA: IBM Corp) [29]. The normal distribution of the data was assessed using the Shapiro–Wilk test. Group comparisons were conducted using either the independent sample *t*-test or Mann–Whitney U test. Categorical data were analyzed using the chi-square test. Results were presented as counts (frequencies), mean (±standard deviation) or median (range: minimal-maximal) values. A *p*-value of <0.05 was considered statistically significant for all the tests.

## 3. Results

Initially, there were 46 children in the group receiving 0.03% atropine eye drops. Over the 1-year follow-up period, 15 patients withdrew from the study. This comprised seven who were lost to follow-up, two who sought for alternative treatment and six who discontinued due to adverse events. Consequently, the 0.03% atropine group had 31 patients remaining for the final analysis. Forty-one myopic children, corresponding to the 0.03% atropine eye drops group, were included as the control group. Baseline characteristics were comparable among the groups (Table 1).

### 3.1. Changes in Ophthalmic Parameters over 1-Year Follow-Up

After 1-year follow-up, the changes in SE and AL were significantly lower in the 0.03% atropine eye drops group compared to the control group (Table 2). In the 0.03% atropine eye drops group, the change in SE was notably lower at −0.34 (0.44) D compared to −0.60 (0.50) D in the control group (*p* = 0.024). Similarly, the change in AL was significantly reduced, measuring 0.19 (0.17) mm in the 0.03% atropine eye drops group compared to 0.31 (0.20) mm in the control group (*p* = 0.015).

No statistically significant differences were observed in IOP, CCT and LT between the groups over the 1-year follow-up period (*p* > 0.05). However, it is noteworthy that the increase in ACD was significantly higher in the 0.03% atropine eye drops group compared to the control group: ACD increased by 0.03 mm in the 0.03% atropine eye drops group in contrast to 0.01mm in the control group (*p* = 0.015).

**Figure 1:**
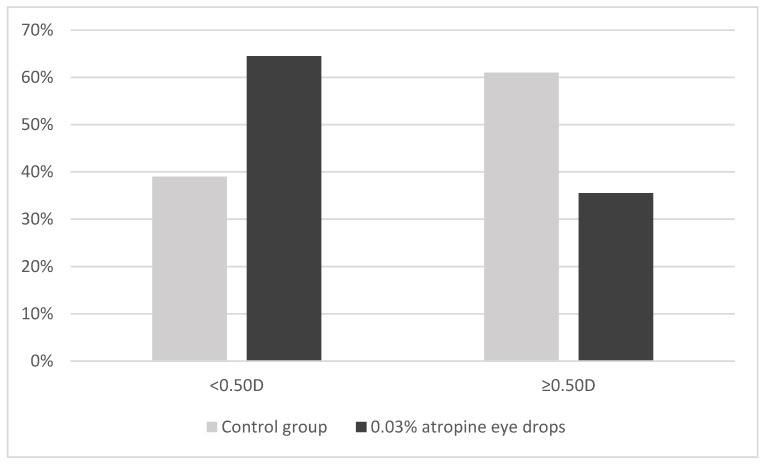
Distribution of change in spherical equivalent (<0.50 D or ≥0.50 D) among control and 0.03% atropine groups over 1-year follow-up. Y-axis represents the proportion of children in each group.

During the 1-year follow-up, 39.0% (16 patients) of subjects in the control group and 64.5% (20 patients) in the 0.03% atropine eye drops group showed SE progression of less than 0.5 D; conversely, 61% (25 patients) in the control group and 35.5% (11 patients) in the 0.03% atropine group showed myopia progression of 0.5 D or more (*p* = 0.032) (Figure 1).

### 3.2. Tolerability of 0.03% Atropine Eye Drops

In the 0.03% atropine eye drops group, adverse events were reported by 13 out of 37 patients, accounting for 35% of the cohort. Among these patients, six individuals, constituting 16.0% of the group, discontinued the use of the 0.03% atropine eye drops due to these events. The most commonly reported adverse reactions were photophobia, documented in 10 patients (27.0%) and blurred near vision, reported by three patients (8.0%). Additionally, one patient developed a viral upper respiratory tract infection followed by a persistent cough during the second month of treatment. Despite efforts to ascertain the cause of the prolonged cough, the underlying etiology remained unclear. Although, upon discontinuation of the 0.03% atropine eye drops, the cough gradually resolved. It is important to note that none of the adverse reactions reported were classified as severe.

## 4. Discussion

In this study the impact of 0.03% atropine eye drops on myopia progression and ocular biometric parameters in a real-world clinical setting among a Caucasian population over a 1-year period was investigated. The findings of this study suggest that 0.03% atropine eye drops significantly controlled myopia progression, as evidenced by changes in SE and AL over the 1-year follow-up. With the administration of 0.03% atropine eye drops, a mean change in SE of −0.34 (0.44) D/year was observed, which was significantly lower compared to −0.60 (0.50) D/year found in the control group. Furthermore, the change in AL was 0.19 (0.17) mm in the 0.03% atropine group, compared to 0.31 (0.20) mm in the control group (*p* = 0.015). Notably, 64.5% of patients in the 0.03% atropine group showed progression of less than 0.5 D/year, compared to 39.0% in the control group (*p* = 0.032).

The present study findings are consistent with previous research indicating the efficacy of low-concentration atropine eye drops in controlling myopia progression. For instance, the LAMP phase 2 study conducted in Hong Kong assessed the efficacy and safety of 0.05%, 0.025% and 0.01% atropine eye drops compared to a placebo [30]. This study demonstrated that the effectiveness of treatment was concentration-dependent, with 0.05% atropine considered optimal concentration after 2 years. Another study conducted in Australia, which included patients of mixed ethnicities, proved the efficacy of 0.01% atropine eye drops over a 1-year period: the mean changes in SE and AL from baseline were −0.31 D and 0.16 mm, respectively, in the 0.01% atropine group compared to −0.53 D and 0.25 mm in the placebo group (*p* ≤ 0.01) [23]. The efficacy observed in that study for 0.01% atropine eye drops in terms of changes in SE and AL is comparable to the findings of the present study with 0.03% atropine eye drops. A study conducted in Spain showed that 0.01% is safe and effective for myopia progression control in a European population after 5 years of use [31]. The results of another 5-year randomized controlled study in Spain concluded that the annual myopia progression rate was −0.14 (0.35) D in the 0.01% atropine group compared to −0.65 (0.54) D in the control group (*p* < 0.01) [15]. A 2-year study by Wang et al. [32] involved Chinese children aged 6–14 years with SE refraction ranging from −1.25 to −6.0 D. They analyzed changes in ocular biometrics in children using 0.02% or 0.01% atropine eye drops and compared them with a control group. Over the 2-years, the changes in SE were −0.81 ± 0.52 D, −0.94 ± 0.59 and −1.33 ± 0.72 D and changes in AL were 0.62 ± 0.29 mm, 0.72 ± 0.31 mm and 0.89 ± 0.35 mm in the 0.02%, 0.01% atropine groups and control group, respectively (*p* < 0.05). This study found that 0.02% atropine showed superiority compared to 0.01% atropine. Interestingly, a multicenter randomized clinical trial conducted by Zadnik et al. [33] showed controversial results. They observed that 0.02% atropine eye drops did not significantly increase the proportion of participants’ eyes exhibiting less than 0.50 D myopia progression from baseline compared to placebo, nor did it significantly reduce myopia progression over a 3-year period. Although, in the same study, the use of 0.01% atropine increased the proportion of eyes showing less than 0.50 D myopia progression after 3 years of treatment compared to placebo. Furthermore, in that study, 0.01% atropine eye drops led to a decrease in both SE and AL changes compared to placebo group over the same period.

However, findings from a 2-year randomized clinical trial involving multiethnic children in the USA, aged between 5.1 and 12.9 years, with a mean SE of −2.83 (1.10) D and mean AL of 24.4 (0.8) mm, do not endorse the efficacy of 0.01% atropine eye drops in controlling myopia progression [16]. Another prospective observational study, encompassing data from the ATOM1 (Atropine for the Treatment of Myopia 1) and ATOM2 (Atropine for the Treatment of Myopia 2) trials included adults from the ATOM1 study (comparing 1% atropine to placebo; 1999 through 2012) and the ATOM2 study (comparing 0.01% atropine to 0.1% and 0.5%; 2006 through 2012) [17]. Across these cohorts, the use of atropine eye drops ranging from 0.01% to 1.0% over a period of 2 to 4 years, commencing with patients at around nine years of age with moderate myopia, did not result in significant differences in final refractive errors observed 10 to 20 years post treatment. These findings underscore the ongoing uncertainty surrounding the long-term efficacy of atropine eye drops for myopia control.

This study also analyzed changes in IOP, ACD, LT and CCT. No statistically significant effects on IOP, LT and CCT were observed. However, a significant increase in ACD was found in the group receiving 0.03% atropine eye drops compared to the control group. This observation in consistent with the findings of a study conducted in Western Australia, which investigated the effect of 0.01% atropine eye drops on controlling myopia progression. In that study, the 0.01% atropine group exhibited a higher increase in ACD compared to the placebo group at 2 years follow-up, with measurements of 0.044 mm and 0.026 mm, respectively (*p* = 0.018) [23]. In addition, another study conducted in Spain showed that the use of 0.01% atropine eye drops is associated with a longer ACD [31]. Research suggests that ACD increases when the pupils are dilated [34,35]. Although we did not directly measure the pupil size in our study, the statistically significant increase in ACD observed in the atropine group, compared to the control group, suggests a significant pupil-dilating effect of 0.03% atropine eye drops. In contrast to the present study findings, a study performed in China by Wang and colleagues [32] did not find a significant difference in the change of ACD between the 0.01%, 0.02% atropine eye drops and control groups. Additionally, findings from the LAMP study suggested a decrease in ACD among individuals using atropine eye drops of varying concentrations, although these changes did not reach statistical significance [36].

In the present study, 35% (13 patients) experienced adverse events such as photophobia and blurred near vision in the 0.03% atropine eye drops groups, but none of these events were considered severe. During the second month of treatment, one patient developed a persistent cough subsequent to a viral upper respiratory tract infection. Despite uncertainty regarding the exact cause of the prolonged cough, discontinuation of the 0.03% atropine eye drops resulted in its resolution. While this association implies a potential link between the atropine eye drops and the cough, definitive causality cannot be established based solely on this observation. Further investigation, including consideration of other possible contributing factors and consultation with a family physician, could have been beneficial to fully understand the possible relationship between the atropine eye drops and patient’s symptoms. In line with previous research on myopia, atropine therapy often leads to commonly reported adverse events such as photophobia and blurred near vision [37]. A retrospective analysis conducted in Germany by Kaymak et al. [38] revealed that nearly 17.0% of children treated with 0.01% atropine reported side effects such as burning eyes following drop instillation, pupil dilatation, photophobia or eye redness. Another study evaluating the impact of 0.01% and 0.05% atropine eye drops on the ocular surface in young myopic adults concluded that 0.05% atropine has a significant yet temporary effect on the tear film and 0.01% atropine has minimal impact on the ocular surface [13]. In a study by Medghalchi et al. [39], four patients (16%) in the 0.1% atropine group experienced headaches compared to none in 0.01% group (*p* = 0.032), while 11 patients (44%) in the 0.1% atropine group had photophobia compared to four patients (20%) in the 0.01% atropine group (*p* = 0.001); additionally, 11 patients (44%) in the 0.1% atropine group experienced blurred vision, while none in the 0.01% atropine group had this side effect (*p* < 0.001). When comparing these findings to present study, we observe that the use of 0.03% atropine eye drops resulted in a higher incidence of adverse events compared to 0. 01% atropine eye drops but a lower incidence compared to 0.1% atropine eye drops. However, it is important to note that the study by Medghalchi et al. was conducted in Asia. For example, while 0.05% atropine is well tolerated in Asian children, it has been associated with more severe and significant side effects in Caucasian children [20]. Notably, impaired vision or reading difficulties were observed in 63.0% of Caucasian children treated with 0.05% atropine eye drops.

However, this study has several notable limitations. Firstly, ethical considerations prevented the implementation of a randomization process, allowing parents to choose whether to use atropine eye drops or not. Secondly, we encountered challenges in assessing patient compliance with the prescribed treatment regimen, which could impact the final study findings. Furthermore, this study did not investigate the outcomes of discontinuing atropine treatment. A systematic review by Sánchez-Tena et al. [14] concluded that different treatments for myopia progression control have a rebound effect after treatment cessation. Specifically, atropine eye drops tend to show a higher rebound effect compared to optical means. A study conducted in Spain showed that children who discontinued 0.01% atropine eye drops after at least 2 years of treatment and were followed up for the subsequent 3 years experienced a rebound effect, resulting in up to a 3-fold increase in the rate of myopia progression after stopping treatment [15]. The LAMP phase 3 study showed that during the third year, SE progression and AL elongation were faster in patients who discontinued treatment with atropine eye drops after 2 years compared to those who continued treatment with 0.01%, 0.025% and 0.05% atropine for a third year [12]. The rebound effect varied with concentration, but the clinical differences were minimal across the studied concentrations.

When considering the economic aspects of myopia progression control, low-concentration atropine eye drops emerge as a cost-effective and accessible option compared to other methods. A systematic review by Agyekum et al. [40] aimed to evaluate the cost-effectiveness of various interventions for preventing myopia, its progression and its associated complications. This analysis concluded that the use of 0.01% atropine is a cost-effective approach to managing myopia progression. Additionally, another analysis by Agyekum et al. [41] compared 13 different interventions for controlling myopia progression in children, which included pharmacological agents, special contact lenses and spectacle lenses, using a Markov model. This economic evaluation suggested that atropine, particularly at a concentration of 0.05%, and outdoor activity are among the most cost-effective strategies for managing myopia progression. However, the number of economic evaluations for myopia progression control remains limited, highlighting the need for further analysis in this area.

The present study revealed that although patients using 0.03% atropine eye drops experienced adverse events more frequently compared to those using 0.01% atropine, the 0.03% concentration effectively controlled myopia progression. Based on the results of this study, considering the risk-benefit ratio, 0.03% atropine eye drops could be recommended for managing myopia progression in children.

## 5. Conclusions

This study demonstrates that despite a higher incidence of adverse events, 0.03% atropine eye drops effectively slowed the progression of myopia over the period of 1-year. The findings of the study support the use of 0.03% atropine eye drops for controlling myopia progression in children. However, further studies are needed to investigate different concentrations of atropine eye drops, as well as the long-term effects and rebound effect after discontinuation of the drops.

## Figures and Tables

**Table 1:** Baseline characteristics of the study subjects. Results are presented as counts (percentages), mean (±standard deviation) or median (minimal-maximal) values. *p*-value < 0.05 was considered statistically significant.

Variables	Control Group (n = 41)	0.03% Atropine Eye Drops Group (n = 31)	*p*-Value
Gender (male, n (%))	14 (34.1%)	10 (32.3%)	0.866
Age at the study’s commencement (years)	10 (6–12)	10 (6–12)	0.789
Self-reported age at first diagnosis of myopia (years)	9 (5–12)	8 (4–12)	0.615
SE (D)	−2.51 (0.91)	−2.67 (0.98)	0.478
AL (mm)	24.23 (1.13)	24.65 (0.82)	0.083
IOP (mmHg)	17.56 (3.20)	17.43 (2.57)	0.885
CCT (μm)	556.85 (31.95)	548.39 (34.18)	0.284
ACD (mm)	3.86 (0.21)	3.85 (0.19)	0.913
LT (mm)	3.30 (0.15)	3.29 (0.12)	0.771

D: diopter; SE: spherical equivalent; AL: axial length; IOP: intraocular pressure; CCT: central corneal thickness; ACD: anterior chamber depth; LT: lens thickness.

**Table 2:** Changes in ophthalmic parameters over 1-year follow-up. Results are presented as mean (±standard deviation). *p*-value < 0.05 was considered statistically significant.

Variables	Control Group (n = 41)	0.03% Atropine Eye Drops Group (n = 31)	*p*-Value
SE (D)	−0.60 (0.50)	−0.34 (0.44)	0.024
AL (mm)	0.31(0.20)	0.19 (0.17)	0.015
IOP (mmHg)	0.91 (2.13)	0.29 (2.44)	0.393
CCT (μm)	1.18 (5.69)	1.26 (8.28)	0.960
ACD (mm)	0.01 (0.04)	0.03 (0.03)	0.015
LT (mm)	−0.0003 (0.05)	−0.01 (0.04)	0.290

D: diopter; SE: spherical equivalent; AL: axial length; IOP: intraocular pressure; CCT: Central corneal thickness; ACD: Anterior chamber depth; LT: Lens thickness.

## Data Availability

The data that support the findings of this study are available upon request from the corresponding author, D.S.
